# Impedance Spectroscopy for the Non-Destructive Evaluation of ***In Vitro*** Epidermal Models

**DOI:** 10.1007/s11095-014-1580-3

**Published:** 2014-12-03

**Authors:** F. Groeber, L. Engelhardt, S. Egger, H. Werthmann, M. Monaghan, H. Walles, J. Hansmann

**Affiliations:** 1 Project Group Regenerative Technologies in Oncology, Fraunhofer Institute for Interfacial Engineering and Biotechnology (IGB), Roentgenring 11, Wuerzburg, 97070 Germany; 2 Department of Cell and Tissue Engineering, Fraunhofer Institute for Interfacial Engineering and Biotechnology (IGB), Nobelstr. 11, Stuttgart, 70569 Germany; 3 Chair Tissue Engineering and Regenerative Medicine, University Hospital Wuerzburg, Roentgenring 11, Wuerzburg, 97070 Germany

**Keywords:** alternative test method, impedance spectroscopy, non-destructive testing, reconstructed human epidermis

## Abstract

**Purpose:**

Reconstructed human epidermis (RHE) is standardly used for the risk assessment of chemical compounds. However, analysis is dependent on invasive methods such as histological processing or 3-(4,5-dimethylthiazol-2-yl)-2,5-diphenyltetrazolium bromide (MTT) staining.

**Methods:**

As an alternative, we have developed a non-destructive technology to analyze the integrity of epidermal equivalents based on impedance spectroscopy. RHEs were generated and impedance spectra were recorded. from these spectra, we extrapolated electrical characteristics such as the capacitance and the ohmic resistance. Furthermore, the measurable electrical parameters were used to quantify the effects of mechanical and chemical disruption of the epidermal integrity.

**Results:**

A fully matured RHE exhibits typical impedance spectra in a frequency ranging between 1 Hz and 100 kHz, which is comparable to the spectra of freshly isolated human epidermal biopsies. We could show that, during RHE maturation, these characteristics change significantly. Thus, capacitance and ohmic resistance can be employed as a criterion for the quality control of skin equivalents. Additionally, our application of impedance spectroscopy reveals sufficient sensitivity to detect a transient decreased ohmic resistance caused by 2-propanol, which is classified as a non-irritant by MTT assays.

**Conclusion:**

These results indicate that impedance spectroscopy can be employed as a non-destructive complementary method to assess mild irritative effects, which is currently not possible.

## Introduction

The epidermis is the outermost layer of the integumentary system and serves as the first barrier that protects the human body from external harmful agents and prevents dehydration due to water loss (1). Hence, the epidermis attracts much focus in the assessment of chemicals and agents that can come into contact with skin. Traditional experiments include various animal models or human *in vivo* studies (2,3) but as an ethical alternative, an in-vitro-generated reconstructed human epidermis (RHE) is proposed (4). RHE recapitulates the cellular and structural properties of the human epidermis, composed of an outer corneous layer and a proliferating basal layer (5). Appreciation of this mimicry of the human epidermis has led to RHEs being employed for several applications in dermatological research ranging from melanoma research (6), the assessment of skin corrosion (7,8), skin irritation (9,10), and the penetration of chemical agents (11).

Histological analysis allows depiction of the RHE architecture but does not provide information regarding the functionality of this tissue equivalent. When employing colorimetric cell viability assays to evaluate the viability of RHE, metabolically active cells contribute to the readout of the assay, wherein the majority of this metabolic contribution is derived from the basal layer as the outer epidermal layers have a significantly reduced metabolism (12). Thus, the effect of a chemical on higher stratified layers cannot be investigated using these techniques. A method that is complementary to RHE viability, which demonstrates the impact of a chemical to the stratum corneum (SC), could allow a more robust classification of the test substance, e.g. a distinction between mild and non-irritants.

The interaction of agents with the SC affects the epidermal barrier (13), and this is a critical consideration in the assessment of RHE. High electrical resistance reflects a strong intact epidermal barrier. A standard output reading for the electrical characterization of cells, cell layers, and tissues is the transepithelial electrical resistance (TEER) (14,15). Usually, the TEER value is obtained by applying a sinusoidal current $\underset{\bar{\mkern6mu}}{I}$ *(f)* [A], and measuring the resulting voltage $\underset{\bar{\mkern6mu}}{U}$ *(f)* [V]. The impedance $\underset{\bar{\mkern6mu}}{Z}$ *(f)* [Ω] can then be determined according to

1 $$ \underset{-}{Z}=\frac{{\underset{-}{U}}_{(f)}}{{\underset{-}{I}}_{(f)}} $$

where $\underset{\bar{\mkern6mu}}{Z}$ *(f)* [Ω] is a complex number. The real component of the impedance value $\underset{\bar{\mkern6mu}}{Z}$ *(f)* [Ω] at a specific frequency in relation to the tissue surface area is the TEER value. However, as impedance is only determined at one frequency, critical information is lost. When measuring at different frequencies, a generated impedance spectrum can be depicted in a Bode plot composed of the amplitude *|* $\underset{\bar{\mkern6mu}}{Z}$ *(f)|* [Ω] and phase angle [°] of $\underset{\bar{\mkern6mu}}{Z}$ *(f)* which allows one to identify electrical elements of the sample. These electrical characteristics can be quantified by employing an equivalent circuit and a mathematical model. Hence, we hypothesize that impedance spectroscopy (IMPS) is a superior method to evaluate the electrical properties of RHE. Here, IMPS is employed to investigate the development of RHEs over time and the response of RHEs to different mechanical and chemical treatments.

## Materials and Methods

### Cell Isolation and Culture

Human epidermal keratinocytes (hEK) were isolated from foreskin biopsies obtained from juvenile donors aged between 1 and 3 years under informed consent according to ethical approval granted by the ethical committee of Landesärztekammer Baden-Württemberg (IGB_ZS_F-2012-078). After washing with phosphate buffered saline (PBS) (Life Technologies; Carlsbad, CA, USA), the biopsies were minced into pieces of approximately 5 mm^2^ and treated with dispase (2 U/mL; Life Technologies) at 4°C for 15 h to digest the basal membrane at the dermal-epidermal junction. Following mechanical removal, the epidermis was incubated in 0.05% Trypsin/EDTA buffer (Life Technologies) at 37°C for 5 min. This enzymatic reaction was halted by the addition of 10% fetal calf serum (Life Technologies) and a hEK suspension was obtained by disrupting the digested epidermal fragments by repeated re-suspension. After centrifugation, the resultant cell pellet was gently re-suspended in EpiLife® medium supplemented with Human Keratinocyte Growth Supplements and 1% Penicillin/ Streptomycin (all from Life Technologies) and cultured in a humidified incubator at 5% CO_2_ and 37°C.

### Isolated Epidermal Samples and Reconstructed Human Epidermis

Isolated human epidermis (IHE) was obtained from female adult donors following cosmetic surgery. The use of skin from adults was performed according to the previously mentioned ethical approval. For separation of the dermis from the epidermis, underlying adipose tissue was removed and biopsy pieces in a size of 2.5 cm^2^ were incubated in dispase (2 U/mL; Life Technologies) at 4°C for 15 h. After incubation, the epidermis was removed gently, mounted on the plastic frames of 12-well transwell inserts (Greiner Bio-One GmbH; Frickenhausen, Germany) and fixed with parafilm® m sealing film (Brand GMBH + CO KG; Wertheim, Germany).

RHEs were generated using a protocol published by Poumay (5). Briefly, 12-well transwell inserts (Greiner Bio-One GmbH) were seeded with 5 × 10^5^/cm^2^ hEK suspended in EpiLife® medium supplemented with Human Keratinocyte Growth Supplement (both from Life Technologies) and 1.5 mM calcium chloride. After 24 h, the cell culture medium was exchanged with EpiLife® medium supplemented with Human Keratinocyte Growth Supplement, 1.5 mM calcium chloride and 50 μg/mL Vitamin C (all from Sigma-Aldrich; St. Louis, USA) and culture conditions were adjusted to create an air-liquid interface for 12 days. During culture, the cell culture medium was renewed three times a week.

### Impedance Measurement

**Fig. 1:**
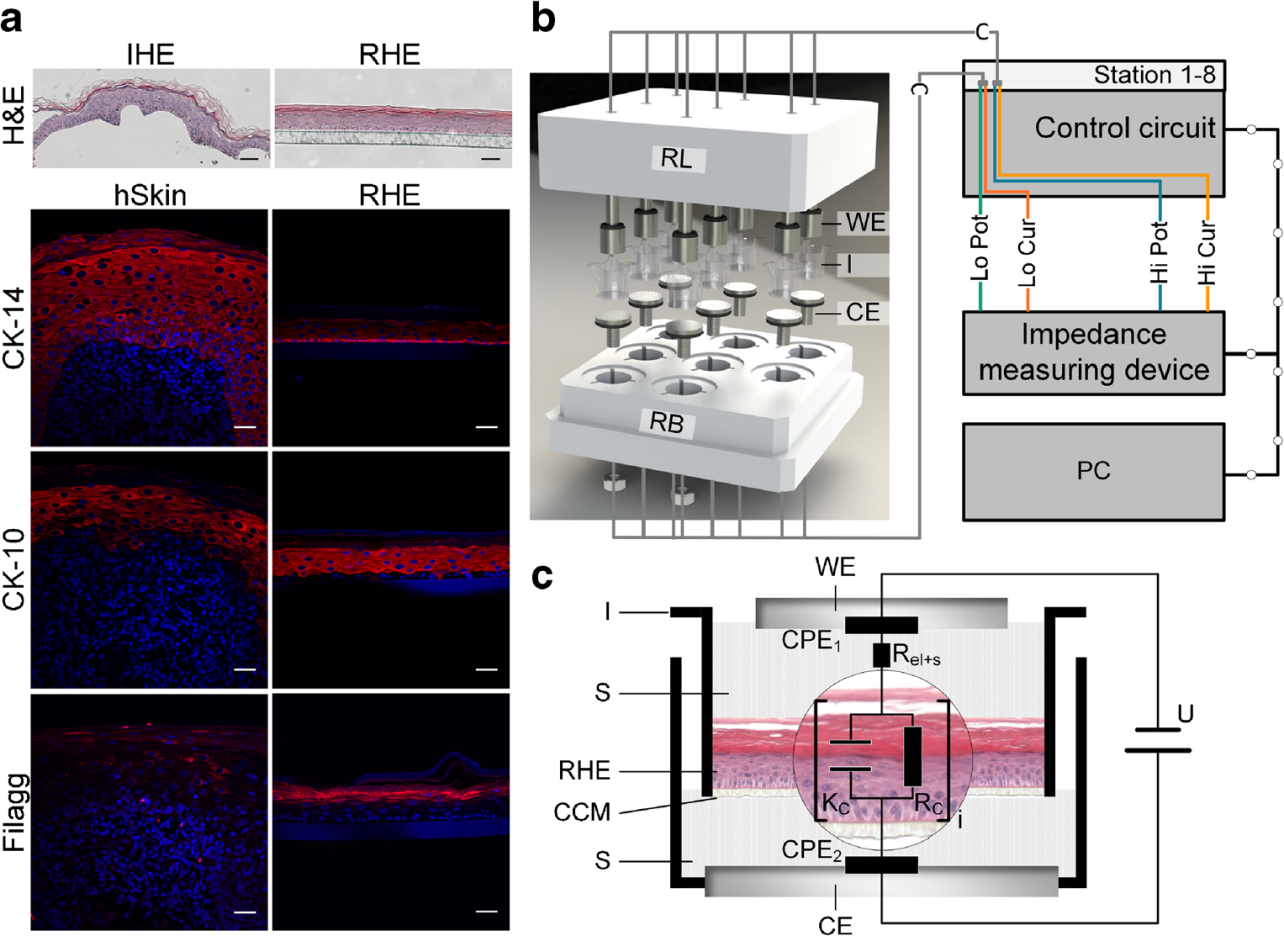
Experimental setup for the impedance measurement of reconstructed human epidermis (RHE). (**a**) Histological characterization of RHE (*right panel*) with isolated human epidermis (IHE) and human full thickness skin (hSkin) (*left panel*). General morphological structure was analyzed using hematoxylin & eosin (H&E) staining. In addition, the epidermal differentiation was evaluated by immunohistofluorescent (IHF) staining against cytokeratin 14 (CK-14), cytokeratin 10 (CK-10) and Filaggrin. *Scale bars* indicate 50 μm. (**b**) Schematic drawing of the measuring system. Eight standard transwell inserts (I) were positioned between a working (WE) and a counter electrode (CE) in a bioreactor system that provided sterile conditions and exact positioning. The electrical impedance of eight samples could be determined consecutively using a commercially available impedance measuring device in combination with a custom-made control circuit. Measurement settings were programmed via a custom-made user interface on a personal computer. (**c**) Detailed view of one measurement chamber with a RHE on a transwell insert (I) with a cell culture treated membrane (CCM) as support between a working (WE) and a counter electrode (CE). The space between the electrodes and the RHE was filled with a conductive saline solution (S) with physiological osmolarity (KBM™-2; PromoCell). The measuring setup was described as an equivalent circuit of two constant phase elements (CPE_1_, CPE_2_), the electrical resistance of the electrodes and the saline solution (R_el+s_) and the electrical properties of the RHE, which includes integral multiples (*n*) of a parallel interconnected capacitor (C_C_) and resistor (R_C_).

IHEs and RHEs were placed into slots between a working and a counter electrode in a sterilized measuring chamber (Fig. 1b). Grooves ensured correct mounting of cell culture inserts in one specific orientation, and thus, a consistent geometrical assembly for multiple experiments. The space between the sample and the counter electrodes was occupied with 1*.*5 ml Keratinocyte Basal Medium 2 (KBM™-2, PromoCell GmbH; Heidelberg, Germany). The inserts were filled with 750 μl KBM™-2 (PromoCell) and the measuring chamber was closed with the electrodes connected to a control circuit. The control circuit connected the impedance spectrometer to one of the eight slots according to the selected parameters. For each slot, impedance was measured over a frequency range from 1 Hz to 100 kHz at 40 logarithmically distributed sampling points. To record the impedance spectra of $\underset{\bar{\mkern6mu}}{Z}$ *(f)* from biological samples, a sinusoidal electric current $\underset{\bar{\mkern6mu}}{I}$ *(f)* [A] was generated and the potential difference $\underset{\bar{\mkern6mu}}{U}$ *(f)* [V] was measured by an impedance spectrometer LCR HiTESTER 3522–50 (HIOKI E.E. Corporation; Ueda, J). A custom-made user interface, programmed in LabVIEW (National Instruments; Austin, USA), calculated $\underset{\bar{\mkern6mu}}{Z}$ *(f)* according to Eq. 1.

### Mechanical and Chemical Treatment of the Reconstructed Human Epidermis

RHEs were injured locally in the middle of the model using a biopsy punch with a round shaped blade, 2 mm in diameter (punch area = 3.14 mm^2^) (Stiefel Laboratories; Triangle Park, NC, USA). Following this, the epidermal layer inside the inflicted wound was carefully removed with tweezers. In another set of models, the partial abrasion of the SC was simulated by tape stripping. To achieve such abrasion, a piece of double-sided adhesive tape, approximately 0*.*8 cm in diameter, was fixed on the top end of a 4 cm long sterilized plastic cylinder. The adhesive tape on the cylinder was then gently pressed, perpendicularly onto the RHEs and peeled off carefully. In total, this procedure was repeated twice with a new adhesive tape to strip further strands of the corneous layer.

To evaluate the effects of chemical substances on the impedance of fully matured RHEs (day 12 of culture), a batch of RHEs was divided into three experimental groups. Only models with an ohmic resistance of at least 600 Ω were considered in the study. Subsequently, 47 μl of test solutions were applied to the RHEs and homogenously distributed over the entire dorsal surface of the RHEs. The first experimental group was subjected to PBS, the second to 5% sodium dodecyl sulfate (SDS) and the third to 2-propanol (both from Sigma-Aldrich). After 35 min incubation at 37°C, the test solutions were removed and the RHEs were washed eight times with 600 μl PBS. Following this, their impedance was measured and the RHEs were cultured in cell culture medium at 37°C and 5% CO_2_. After 42 h, the impedance values were again recorded and viability was evaluated using a quantitative 3-(4,5-dimethylthiazol-2-yl)-2,5-diphenyltetrazolium bromide (MTT) assay (16). Briefly, all RHEs were placed in 380 μl of 0.1 mg/ml MTT in PBS for 3 h. The MTT was then dissolved by incubating the RHE in 3.8 ml 2-propanol (Sigma-Aldrich) for 12 h at 4°C and thereafter quantified by measuring the optical density at 570 nm in a spectroscopic reader.

### Immunochemical and Histological Staining

Full thickness skin biopsies, IHEs and RHEs were fixed in Histofix® (Carl Roth GmbH; Karlsruhe, Germany) and embedded in paraffin. For analysis of general morphological architecture, 3 μm cross sections were stained with hematoxylin & eosin (H&E). Bright-field images were obtained by a Zeiss Axiovert 200 microscope (Carl Zeiss MicroImaging GmbH; Göttingen, Germany). For immunohistofluorescent (IHF) staining, 3 μm cross sections were hydrated and subjected to antigen retrieval using tris- ethylenediaminetetraacetic acid buffer. After blocking unspecific binding with goat serum and bovine serum albumin, 500 μl of a primary antibody solution [Cytokeratin 14 (CK14), 1:500 (Sigma-Aldrich); Cytokeratin 10 (CK10), 1:500 (Dako; Glostrup, Denmark) and Filaggrin, 1:50 (Biomol GmbH; Hamburg, Germany)] were applied and incubated for 12 h at 4°C. The primary antibody solution was removed and secondary antibodies coupled with Alexa Fluor® 594 (Dako; Glostrup, Denmark) were applied [CK14, IgG1 anti rabbit (Sigma-Aldrich); CK10 and Filaggrin IgG1 anti mouse (Sigma-Aldrich)]. After 30 min staining at room temperature, the slides were washed and cell nuclei were counter-stained with 4′,6-diamidino-2-phenylindole (DAPI). Finally, the slides were covered with ProLong® Gold Anti Fade solution (Life Technologies) and enclosed by cover glasses.

### Statistical Analysis

Quantitative data was analyzed for statistically significant differences using a one-way ANOVA employing the Fisher’s least significant difference test. For all statistical tests a *p*-value <0.05 was considered significant.

## Results

### The Reconstructed Human Epidermis Recapitulates The Structure of the Human Epidermis

To demonstrate the capability of RHEs to reflect the human epidermis anatomically, histological cross sections were analyzed employing H&E staining and IHF (Fig. 1a). RHEs were organized as a layered structure with five to eight vital cell layers and a mature SC. Moreover, the viable cell layers could be further divided into one basal layer (stratum basale) with cubical cells, three to four spinous layers with flattened cells (stratum spinosum) and one to three layers containing flattened hEK with granula (stratum granulosum). This histological architecture was comparable to the organization of IHE. Characteristic epidermal differentiation of the RHEs was confirmed by IHF. CK-14; as a marker of the basal keratin network; is predominant in the basal layer and decreases in intensity along the ascending layers. In contrast, CK-10 was not present below the first suprabasal layer. The expression of Filaggrin, as part of the cornified envelop, was limited to the stratum granulosum.

### Electrical Characteristics of Isolated and Reconstructed Human Epidermis can be Described as a Series of a Parallel Circuit Consisting of a Capacitor and Ohmic Resistor

To measure the electrical impedance of RHEs, a novel measurement system was developed (Fig. 1b). Based on the experimental setup, the equivalent circuit diagram as shown in Fig. 1c was applied. It is composed of electrical components attributed to the biological samples and components, which reflect the wiring and interfacial effects at the electrodes’ surfaces. The electrical equivalent circuit is described as:

2 $$ \begin{array}{l}\underset{\bar{\mkern6mu}}{Z}={R}_S+{\underset{\bar{\mkern6mu}}{Z}}_{CP{E}_1}+{R}_{el}+{\underset{\bar{\mkern6mu}}{Z}}_{CP{E}_2}+{\displaystyle \sum_{i=1}^n{\underset{\bar{\mkern6mu}}{Z}}_{Ci}}\\ {}={R}_{el+s}+\frac{2}{K_{CPE}{\left(j\omega \right)}^{N_{CPE}}}+{\displaystyle \sum_{i=1}^n\frac{R_{Ci}}{1+{K}_{Ci}{R}_{Ci}{\left(j\omega \right)}^{N_{Ci}}}}\end{array} $$

The ohmic resistance *R*_*el+s*_ [Ω] accounts for the resistances of the circuit and the resistance of the cell culture medium. In this study, *R*_*el+s*_ was experimentally determined to be 34 Ω. Imperfect characteristics of the electrodes are compensated by two constant phase elements (CPEs) by the parameters *K* [Ss^N^/rad^N^] and *N* [a.u.]. The two CPEs are merged into an apparent CPE with the parameters *K* = 5.8 × 10^−5^ Ss^N^/rad^N^ and *N* = 0.84 [a.u.]. IHEs and RHEs are represented by a series connection of *n* parallel resistor-capacitor-circuits with a non-ideal characteristic of the capacitors. The parameters of the components that were determined by the tissue are *R*_*Ci*_ [Ω], *K*_*Ci*_ [Ss^N^/rad^N^] and *N*_*Ci*_ [a.u.] with *n* ≥ 1. To derive biological relevant parameters from the spectra, a suitable equation (Eq. 2) was fitted to the acquired data points via a least square minimization algorithm (Matlab, Mathworks; Natick, USA).

### The Impedance Spectra of Isolated and Reconstructed Human Epidermis After 9–12 Days of Culture at the Air-Liquid Interface are Comparable and Both Differ Significantly From Cell-Free Membranes and Undifferentiated RHEs

**Fig. 2:**
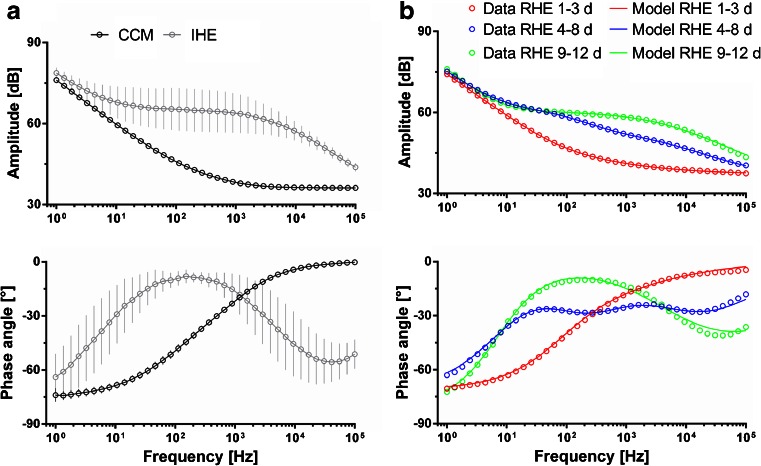
Bode plots of impedance spectra from cell culture treated membranes (CCM), isolated human epidermis (IHE) and reconstructed human epidermis (RHE) at different time points. (**a**) Comparison between mean curves of CCM and IHE. For the IHE 10 samples from five donors were measured. The data for the CCM were obtained from 10 measurements. (**b**) Exemplary data sets of RHE cultured for 1-3 days, for 4-8 days and for 9-12 days at the air-liquid interface. The measured data points for the amplitude of the impedance and the phase shift between voltage and current are depicted in each diagram as circles. *Unbroken lines* indicate the results of the fitting using the model shown in Eq. 2. The data sets of RHE cultured for 1-3 days and 9–12 days at the air-liquid interface could be modeled with one electrical barrier (number of parallel resistor-capacitor-circuits *n* = 1) whereas fitting of RHE cultured for 4-8 days at the air-liquid interface required a second electrical barrier (number of parallel resistor-capacitor-circuits *n* = 2).

The tissue maturation process of RHEs was monitored by time-lapse IMPS. The obtained spectra are depicted as Bode plots (Fig. 2). To estimate the expected domain of the data, the impedance spectra of unseeded cell culture membranes (CCM) and IHEs of different donors were recorded (Fig. 2a). At each data point, mean values and standard deviations of the magnitude and the phase shift of the impedance spectra of 10 CCM and IHE are shown. When comparing the measured data sets of RHEs at different time points, the spectra showed a specific curve progression over time. Based on the measured data, the process could be divided into 3 phases beginning from the exposure of RHEs to the air-liquid interface (Fig. 2b); an early phase up to 3 days, an interim phase between 4 and 8 days, and a late phase from 9 up to 12 days. During the early phase, the spectra of RHEs were similar to the spectra of the cell free cell culture membranes. In the interim phase, the magnitude and the phase shift of RHEs differed from those of the CCM and IHEs. In the late phase, Bode plots of RHEs were in the range of IHE Bode plots. An impedance spectrum can be described by the number of frequencies where a change of the slope of the magnitude and a shift in the phase angle are measured. While in the early phase, such a corner frequency appeared only once at approximately 110 Hz, two corner frequencies at around 100 and 1200 Hz could be seen in the spectra of the late phase. The unsteady waveform of magnitude and phase shift at the interim phase indicated more than three corner frequencies. In addition to the measured data points, modeled spectra are shown. The varying complexity of the spectra resulted in different numbers (*n*) for the required resistor-capacitor-circuits (Eq. 2), when applying the equivalent circuit. The early phase and the late phase could be sufficiently fitted, using a one resistor-capacitor-circuit. For the interim phase, two independent resistor-capacitor-circuits were necessary.

### Epidermal Tissue Formation Causes a Decrease in Electrical Capacitance and an Increase of the Ohmic Resistance

**Fig. 3:**
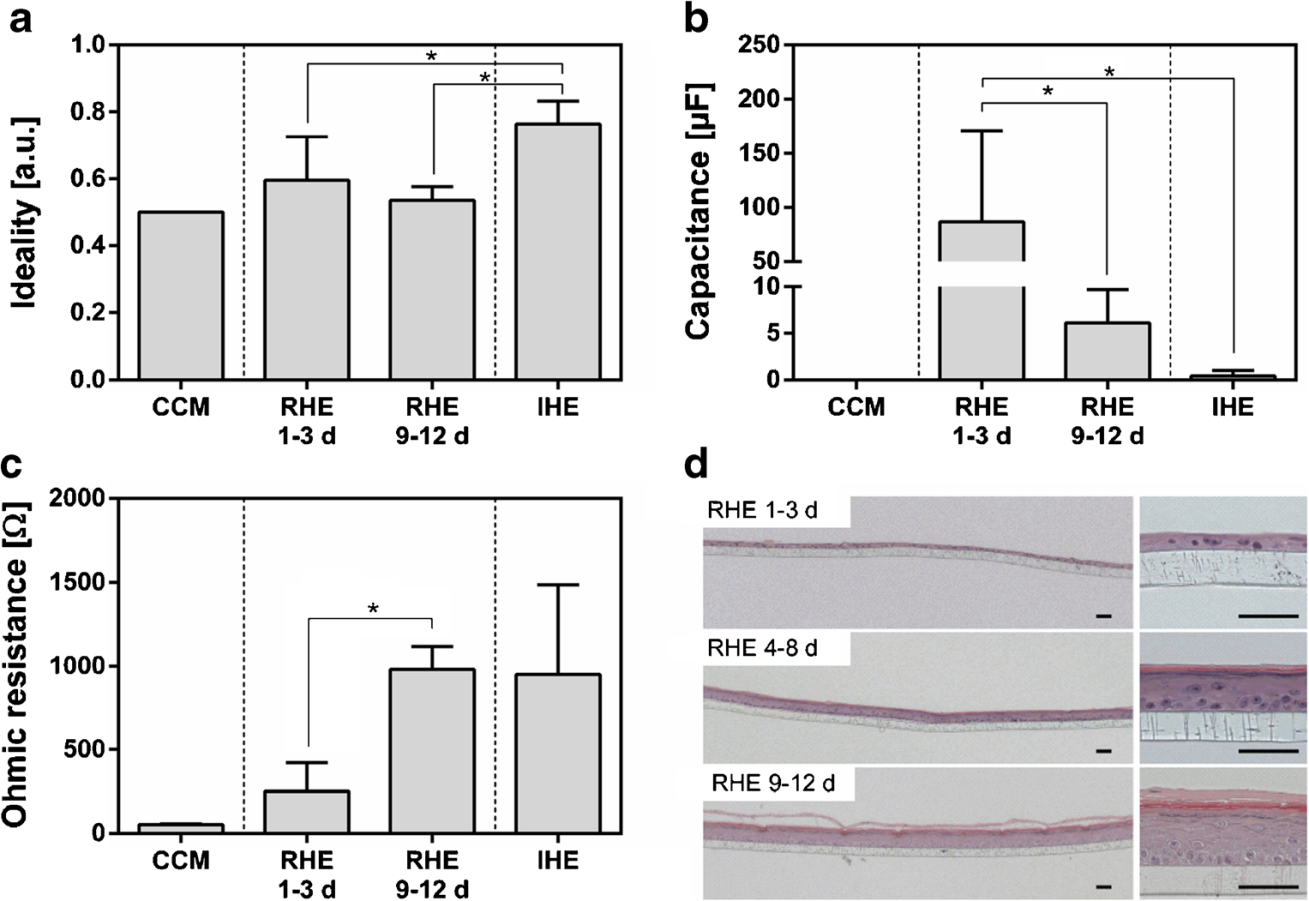
Mathematical modeling and histological cross sections of reconstructed human epidermis (RHE) cultured for different times at the air-liquid interface. Depicted are the mean values and standard derivations for the ideality (**a**), the capacitance (**b**) and the ohmic resistance (**c**). Each diagram shows the derived values for the cell culture treated membranes (CCM), RHE that are cultured for 1-3 days (RHE 1-3 d) and 9-12 days (RHE 9-12 d) at the air-liquid interface as well as the values of the isolated human epidermis (IHE). Each experimental group was comprised of 10 samples. *Stars* indicate statistical relevant differences (*p*-value ≤0.5). (**d**) H&E stained histological cross sections of RHE cultured for 1-3 days (RHE 1–3 d), 4-8 days (RHE 4-8 d), and 9-12 days (RHE 9-12 d) at the air-liquid interface. *All scale bars* indicate 50 μm.

Employing the mathematical model (Eq. 2), the cellular components of RHEs and IHEs were characterized by their capacitance, ohmic resistance, and ideality of the capacitor. The range of values for the ideality of the capacitor lay between 0 and 1. Between the ideality of the cell culture membranes and RHEs at the early and late culture phase, no statistically relevant differences could be detected. Comparing RHEs and IHEs, the ideality of IHEs was significantly higher (Fig. 3a). When fitting the measurements of the CCM with this model, the values for capacitance became less than 1 × 10^−19^ Farads (F), which demonstrated a negligible capacitive potential. During the maturation of RHEs, the capacitance dropped significantly from the highest value of 86.8 μF in the early phase to a mean value of 6.11 μF in the late phase. In comparison, the capacitance of IHEs was 0.43 μF and no statistically significant difference between the capacitance of IHEs and that of RHEs of the late phase was detected (Fig. 3b).

The lowest average value of the ohmic resistance was 91.6 Ω, which was calculated for the CCM. This ohmic resistance was only slightly increased to an average of 217 Ω during the early culture phase. During epidermal differentiation, electric resistance increased significantly to a mean value of 980.6 Ω for RHEs in the late culture phase. This value was within the range of the ohmic resistance of IHEs, which had an average ohmic resistance of 951.1 Ω (Fig. 3c).

The shift of the electrical properties of RHEs during the epidermal differentiation could be attributed to architectural changes of the tissue (Fig. 3d). In the early culture phase, hEKs formed a continuous monolayer on the cell culture membrane where all cells exhibited the same morphology with no SC present. After 4 to 8 days of culture at the air-liquid interface, cells in the basal layer began to develop a cubic morphology and hEKs appeared to flatten in higher cell layers. Furthermore, a single cell layer with granula and a thin SC was detectable. In the late culture phase from 9 to 12 days the epidermal differentiation was complete and the thickness of the viable cell layers and the SC increased.

### Ohmic Resistance is a Suitable Parameter to Assess Mechanical and Chemical Trauma

**Fig. 4:**
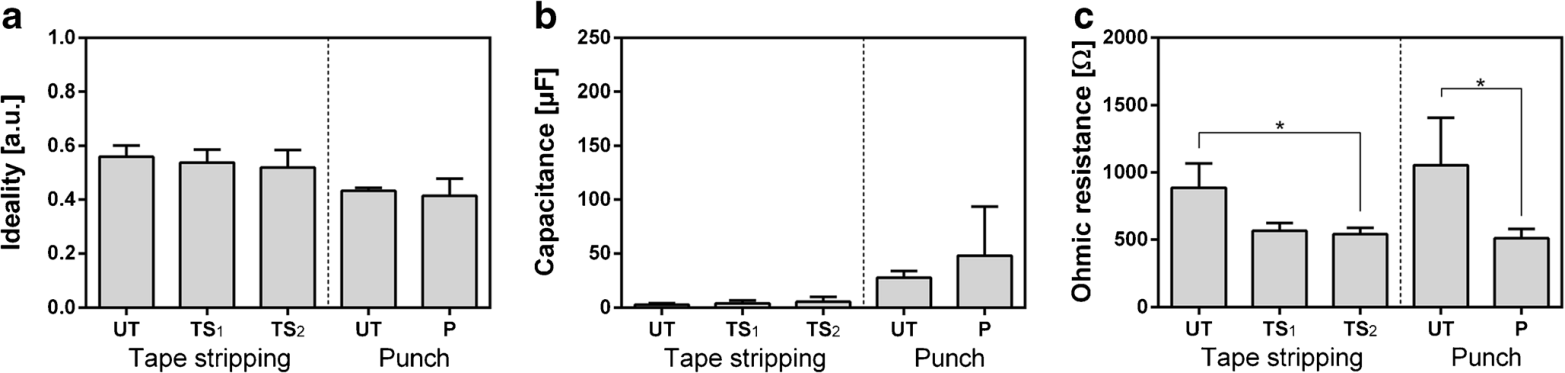
Change of the impedance of reconstructed human epidermis (RHE) cultured for 12 days at the air-liquid interface following mechanical disruption by the repeated removal of the corneous layer by tape stripping (Tape stripping) and the extraction of a full thickness tissue sample using a 3.41 mm^2^ biopsy punch (Punch). The mean values and standard deviations of the ideality (**a**), capacitance (**b**) and ohmic resistance (**c**) were determined of untreated RHE (UT) and RHE that had been subjected to the treatments. In the tape stripping experiments the upper corneous layers were removed twice and the impedance was measure separately after the first (TS_1_) and the second treatment (TS_2_), whereas RHE were punched only once (P). Stars indicate statistical relevant differences (*p*-value ≤0.5). Each group comprises 3 individual samples.

Figure 4 depicts the impact of mechanical disruption of RHEs by removing the upper parts of the corneous layer by tape stripping or applying a local 3.14 mm^2^ circular full thickness defect by a biopsy punch. The ideality of the cellular capacitor and thus its roughness was not changed by the removal of the SC or a full thickness defect (Fig. 4a). Furthermore, the capacitance was not significantly altered due to tape stripping. In contrast, a 3.14 mm^2^ defect appeared to effect an increased capacitance from 27.6 to 48.0 μF (Fig. 4b). However, the most significant changes due to these mechanical treatments were reflected in the ohmic resistance values of RHEs. Tape stripping caused a reduction in resistance, from a mean resistance of 885.8 to 565.6 Ω after the first treatment and to 540.4 Ω after the second iteration. A full thickness defect of 3.14 mm^2^ in diameter decreased the ohmic resistance likewise from 1054.4 to 510.4 Ω (Fig. 4c).

**Fig. 5:**
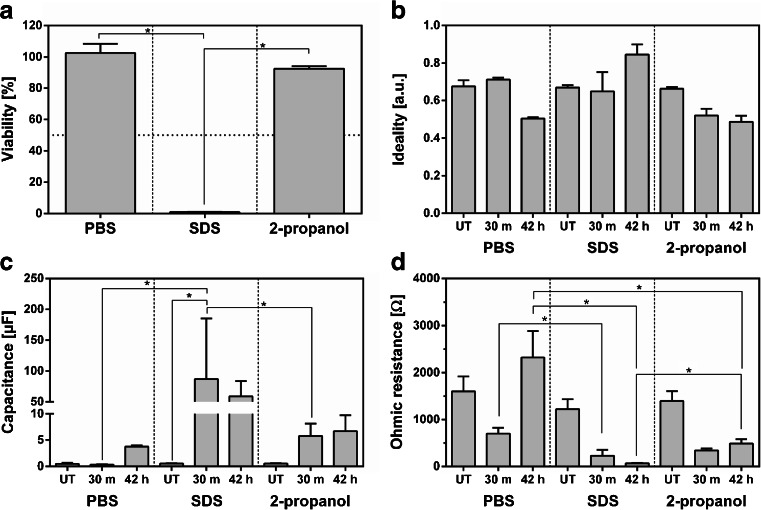
Change of the impedance of reconstructed human epidermis (RHE) cultured for 12 days at the air-liquid interface following chemical treatment with PBS, SDS and 2-propanol. The mean viability of RHE and standard deviation were determined using a quantitative 3-(4,5-dimethylthiazol-2-yl)-2,5-diphenyltetrazolium bromide (MTT) assay in which the viability of the PBS group is set to 100% (**a**). The mean values and standard deviations of the ideality (**b**), capacitance (**c**) and ohmic resistance (**d**) were determined before the application of the test substances (UT), after a 30 min incubation phase followed by eight washing steps (30 m), and after a 42 h recovery phase (42 h). *Stars* indicate statistical relevant differences (*p*-value ≤0.5). Each group comprises 3 individual samples.

Finally, we determined the effect of three different chemical substances on the impedance and viability of RHE (Fig. 5). MTT staining indicated tissue viability and confirmed the irritative properties of SDS as the viability was decreased to 0.98%. In contrast, 2-propanol caused only a small reduction in viability to 87.93% (Fig. 5a). In addition to viability, the impedance of RHEs was measured before the application of the test formulations (UT), directly after application and washing (30 m) and before the MTT assay (42 h). The application of the test substances on RHEs had a minimal effect on the ideality and capacitance of the capacitor (Fig. 5b and c) in general. When treated with SDS, the capacitance of RHEs was increased significantly compared to the other experimental groups (Fig. 5c). However, within the groups and following the application of the test substances, major changes of the ohmic resistance were detectable (Fig. 5d). After application of PBS and a subsequent washing step, the ohmic resistance was decreased from 1603.0 to 698.5 Ω. Following the 42 h recovery time, the resistance recovered to 2324.3 Ω. When SDS was applied to the models, the resistance dropped from an initial value of 1223.5 to 227.2 Ω following the washing steps whereas the resistance was significantly lower than in the PBS group. Interestingly, after the recovery phase, the resistance in the SDS treated RHE did not recover but decreased to 66.8 Ω. In the third group, treatment with non-irritative 2-propanol reduced the ohmic resistance in a range that was comparable to the SDS group from 1394.5 to 340 Ω after the washing step. However, following the recovery phase, the ohmic resistance is 487.3 Ω, which was statistically higher than the recovery in the SDS group.

## Discussion

Here, a system that employs IMPS to investigate RHEs in a non-destructive manner was achieved. In contrast to standard TEER measurements that measure impedance only at a single frequency, usually at 12.5 Hz (17), a robust multi-frequency impedance spectrum was obtained. Thus, more information is acquired. Despite advantages of IMPS, such as easy integration into any experimental setup, IMPS is not yet an established standard method for the characterization of 3D tissue equivalents. Most currently available handheld systems are designed for 2D cell cultures, and do not meet criteria to evaluate highly structured 3D tissues (18,19). In this study, RHEs were composed of multiple cell layers and each cell layer had a specific impact on the electric parameters. By means of mathematical modeling, distinct electrical barriers were represented by parallel resistor-capacitor-circuits. To represent the different phases of RHE-maturation, the number *n* of these circuits was variable. The number of corner frequencies observed in the measured Bode plots (Fig. 2) supports this approach. In the early phase, RHEs had not yet developed a 3D architecture, and thus, could be described with *n* = 1. In the interim phase, additional cell layers were present and a corneous layer began to form. Hence, two electrical barriers (*n* = 2) were present. In the late phase, the corneous layer was strengthened, and its electrical properties were dominant and *n* became 1 again. The equivalent circuit presented here, provides a sufficient degree of freedom to fit the experimentally derived Bode plots during complete 3D epidermis maturation phase, in contrast, to a simple model published for 2D cell cultures (19). Furthermore, in previous equivalent circuits for biological barriers, the capacitors were considered as ideal units. An ideal capacitor shows a typical amplitude decay of −20 dB per decade, which was not present in the acquired Bode diagrams. Therefore, the parameter *N* [a.u.] was introduced to describe the ideality of the biological capacitors.

The equivalent circuit developed for the RHEs corresponded to that of IHEs and skin *in vivo* as reported by Yamamoto and Yamamoto (20). In this study, it was postulated that the apparent impedance of human skin is composed of two distinct electrical barriers; one for the SC and one for the vital cell layers. *In vivo*, the resistance of the SC was considerably higher than the resistance of the vital cell layers (20). Thus, the existence of two distinct layers *in vivo* could not be confirmed experimentally. However, our *in vitro* data supports this hypothesis by the identification of an intermediate phase during the RHE maturation, when the developing corneous layer and the vital cell layers contributed significantly to the apparent impedance.

Based on the parameterized electrical equivalent circuit, the electrical properties of the biological system were derived. The ideality of the capacitor was significantly lower in RHEs than in IHEs. This can be explained by surface roughness. The ventral surface of RHEs is defined by the porous structure of the underlying cell culture membrane. The corresponding capacitances decreased significantly during RHE maturation which can be attributed to the inverse correlation between capacitance and the thickness of a capacitor. This hypothesis is strengthened by the histological analysis of RHEs over the culture time which shows an increasing tissue thickness with advancing maturation (Fig. 3d). In contrast, the ohmic resistance increased during epidermal differentiation. This reflects the increasing electrical impermeability of the SC and the formation of tight junctions in the vital cell layers. Fully developed RHEs showed comparable values for the capacitance and ohmic resistance similar to IHEs. However, the electrical parameters of the IHEs were different to data derived experimentally from *in vivo* and ex vivo measurements. On average, the measured capacitances were generally 10 to 100 fold higher and the ohmic resistance 3 to 8 times lower (21,22). A reason for this can be differences in the chemical composition of the SC between human skin and skin equivalents (23) and the dependency of electrical conductivity on the degree of moisture (21). While *in vivo* or ex vivo studies are typically conducted at the tissue-air-interface, this study was performed in the liquid phase.

In addition to monitoring epidermal differentiation, IMPS can be employed as a live-time non-destructive analysis tool of RHEs. Currently, the barrier function of RHEs is tested by its resilience against strong detergents such as Triton-X (16). Here, we showed that the barrier function of RHEs is impaired by the partial removal of the corneous layer by tape stripping or by a local injury via biopsy punching. A significant drop of the ohmic resistance indicates that IMPS is suitable as an additional non-destructive quality evaluation tool for RHE that allows the assessment before an experiment, and thus, the reduction of false-positive results.

When RHEs are used in the assessment of skin irritants, substances are classified as irritants or non-irritants by their effect on the viability of RHEs and/or the levels of interleukin-1α (IL-1α) (9,12). To test the applicability of IMPS as a complementary endpoint in skin toxicity testing, we compared three different substances with known irritative potential according to the current OECD test guideline (24). In contrast to standard testing procedures, IMPS allows comparison of individual RHEs before and after a treatment. Interestingly, the washing step with PBS alone reduced impedance, which later recovered, indicating a reversible effect. In contrast, SDS caused a significantly stronger and irreversible drop of resistance, which led to an increase of capacitance. This can be explained by a reduction of the capacitor thickness. These findings indicate that IMPS is capable of distinguishing between the effect of strong irritants and non-irritants. It was also shown that non-irritant 2-propanol significantly reduced the ohmic resistance of RHEs, which is contradictory to the readouts of the viability test. MTT based assays assess the viability of only the basal cell layer in RHEs (12). Thus, no direct information of the effect on the outer epidermal layers is gained. These non-viable layers create the major part of the skin barrier, and hence, are of pivotal interest in the assessment of test substances. The impairing reaction of the upper epidermal layers with 2-propanol can be explained by the capability of 2-propanol to dissolve lipids such as free fatty acids in the SC (25). These lipids fill the extracellular space in the SC and contribute significantly to the skin barrier (23,26). These results indicate that together with viability assays IMPS could be used to identify sub-irritative effects on the skin such as a stinging, burning, or itching sensation.

## Conclusion

This study demonstrates that IMPS has the ability to refine RHE-based testing strategies. Such a system could be incorporated as an in-process control and to characterize the barrier functionality of the epidermis. Thus, the quality of RHEs can be ensured in advance of a testing procedure. IMPS can also act as new endpoint for the assessment of skin irritation complementing current viability tests. The additional information regarding the effect of a substance on the tissue integrity allows a better understanding of the toxic potential of the substance. Overall the significance of using IMPS is that it is a non-destructive technique that requires only a short processing time. Furthermore, the parallelization of IMPS measurements compared to other techniques is relatively simple which supports high-throughput testing.

## Abbreviations

CCM: Cell culture treated membrane
DAPI: 4′,6-diamidino-2-phenylindole
H&E: Hematoxylin & eosin
hEK: Human epidermal keratinocytes
IHE: Isolated human epidermis
IHF: Immunohistofluorescent
IMPS: Impedance spectroscopy
KBM™-2: Keratinocyte Basal Medium 2
MTT: 3-(4,5-dimethylthiazol-2-yl)-2,5-diphenyltetrazolium bromide
OECD: Organisation for Economic Co-operation and Development
PBS: Phosphate buffered saline
RHE: Reconstructed human epidermis
SC: Stratum corneum
SDS: Sodium dodecyl sulfate
TEER: Transepithelial electrical resistance
